# Knowledge of HIV transmission and condom use among HIV-positive heterosexual men and women in Guatemala

**DOI:** 10.1186/1758-2652-14-58

**Published:** 2011-12-19

**Authors:** Juan J Delgado Hurtado, Marcela Pineda, Iris Cazali, Carlos Mejía

**Affiliations:** 1 Universidad Francisco Marroquín, School of Medicine, Guatemala City, Guatemala; 2 Infectious Diseases Unit, Roosevelt Hospital, Guatemala City, Guatemala

## Abstract

**Background:**

The prevalence of HIV/AIDS in Guatemala among the general population is 0.79%, and 94% of transmission is directly related to sexual contact. Studies have been conducted on high- prevalence HIV-positive populations (men who have sex with men, commercial sex workers and prisoners). Heterosexual transmission has gained importance in the epidemic in Central America. To our knowledge, no study addressing knowledge of mechanisms of HIV transmission and condom use has been done on HIV-positive heterosexual men and women.

**Methods:**

A closed-ended structured interview that addressed knowledge of mechanisms of HIV transmission and condom use was conducted on 283 heterosexual HIV-positive men (54.8%) and women (45.2%) outpatients who attend the Roosevelt Hospital's Clinic of Infectious Diseases in Guatemala City. Differences between selected characteristics were examined for significance using the Chi-square test. A multiple logistic regression was done to determine socio-demographic variables associated with inconsistent condom use.

**Results:**

Of the interviewed persons, 68.5% were either living with a partner or married, and 94.3% were currently using antiretroviral therapy. Most respondents knew the mechanisms of transmission of HIV. 81.7% and 87.3% reported always using a condom with their regular and casual sexual partner in the past year, respectively. There was no statistically difference in condom use according to the patient's formal education, gender, type of partner (regular or casual)or number of sexual partners. According to the interviewees, 72% of sexual partners in the past year were either HIV negative or of an unknown serostatus. Potentially, these HIV-negative persons are at risk of contracting the virus. Among the main reasons given for not using a condom were: "my partner did not want to use a condom"; and "the condom irritates or makes my partner uncomfortable".

**Conclusions:**

Since no socio-demographic or sexual behavior variable was associated with inconsistent condom use, we recommend intensive and regular condom counselling for every heterosexual HIV-positive outpatient who attends the clinic. Further studies should be done to determine condom use negotiation between partners; and to determine social, interpersonal and psychological factors that might affect the decision to use a condom or not.

## Background

Guatemala is the most populated country in Central America, with almost 14 million inhabitants [1], 3 million of whom live in Guatemala City, its capital [2]. It is estimated that 51% of the country's population live in poverty [3]. Guatemala's population annual growth rate is 2.06% [1], which is higher than that of the rest of Latin America. According to the Human Development Index, it is categorized as a medium-developed country with a current ranking of 131 out of 187 [4].

The prevalence of HIV/AIDS among the general population is 0.79% [1]. It is estimated that 94% of transmission is directly related to sexual contact and that 5% of the new cases are transmitted vertically. The HIV-positive population lives predominantly in urban areas along commercially important roads and migration routes to the United States and Mexico. Most of the cases (80%) have occurred in persons in the range of 15 to 49 years of age. The prevalence of HIV among men who have sex with men (MSM) is 18.3%, among commercial sex workers 1.09% and among prison inmates 3.24% [1].

The number of HIV cases in women is increasing. According to information provided by the two HIV clinics located in Guatemala City (Luis Angel Garcia, San Juan de Dios Hospital and Infectious Diseases Clinic, Roosevelt Hospital), in 2004, 74% of new HIV cases were women without any risk factor other than having sexual relations with their regular partner [5]. This feminization of the epidemic is probably a result of having sex with infected men who, in their turn, have had extramarital sexual relations with HIV-positive people [5].

To provide effective measures that diminish HIV transmission, it is important to study the knowledge of mechanisms of HIV transmission, sexual behaviour and condom use among heterosexual HIV-positive patients. In some countries, this type of transmission has been increasingly examined. In Guatemala, there has been some research done on condom use and knowledge of HIV transmission in sex workers [6] and MSM [7].

According to a study done on MSM, 82.3% of respondents expressed consistently using a condom with their casual partner in the previous month, while 62.8% reported consistently using condoms with their regular partner in the previous month. In total, 80.1% of the sample had used a condom in their most recent sexual relation. This study also addressed knowledge of mechanisms of HIV transmission. Of the respondents, 21.9% thought mutual faithfulness was a method of preventing transmission of HIV [7]. A study done on female sex workers reported that 78.8% and 96.1% of the sample expressed using a condom consistently with their casual and regular sex clients, respectively, in the previous month [6].

A study that examined the impact HIV voluntary counselling and testing had on self-reported behavioural risk (in the previous three months) of people living with HIV (PLHIV) found that it resulted in a modification of risky behaviour. Initially, before being counselled and diagnosed with HIV; 12.19% (5) of the sample (41 persons) stated that they had never had unprotected sexual relations in the prior three months. On the follow up visit, three months after counselling and diagnosis, 73.17% reported never having unprotected sexual relations in the prior three months [8]. This difference was statistically significant.

Heterosexual transmission has gained importance in the epidemic in Central America. In some countries, this type of transmission has been increasingly examined, but in Guatemala to our knowledge, few studies have been done on heterosexual people. Therefore, the objectives of this study were to describe the socio-demographic characteristics of the sample, to determine the knowledge of the mechanisms of HIV transmission, to determine condom use among heterosexual PLHIV, and to identify variables associated with inconsistent condom use. This can help generate more focused counselling for non-condom-adherent heterosexual PLHIV.

## Methods

This cross-sectional descriptive study was conducted in 2010 at the Roosevelt Hospital's Clinic of Infectious Diseases, an outpatient clinic, in Guatemala City, under the approval of the Roosevelt Hospital Ethics' Committee and the Research Department of Universidad Francisco Marroquín.

Persons eligible to participate in this study were PLHIV, both men and women, attending the clinic; they were 18 years or older, sexually active in the past year and had been diagnosed with HIV between one and five years earlier by double ELISA or Western Blot. Subjects who had same-sex relations in the preceding year, worked as commercial sex workers or had been imprisoned were excluded from the sample.

The subjects were approached in the clinic's waiting area. Before being interviewed, they had to sign an informed consent form. A closed-ended, structured survey interview was conducted on those who were eligible according to the selection criteria. The survey included socio-demographic characteristics, mechanisms of transmission of HIV, and sexual behaviour, including condom use and reasons for not using a condom. The interview was held in a private counselling room within the clinic by an interviewer previously trained by the main investigators.

Interviewees were presented with hypothetical situations, asked if the situations could lead to the transmission of HIV/AIDS, and were questioned on ways to prevent HIV transmission. The answers were either "yes", "no" or "I do not know". They were asked to classify their condom use consistency (based on a Likert-type scale) in the past six months and during the past year with their regular and casual partners (if they had any). Condom use in the most recent sexual relation was questioned. Persons were asked if they had any reason for not using a condom in the past year through a closed-question approach, allowing multiple reasons. If another reason not included in the list was mentioned, it was added in the data.

For the analysis, Epi Info 2000 was used. Differences between selected characteristics were examined for significance using the Chi-square test. A multiple logistic regression was done to determine the socio-demographic characteristics associated with inconsistent condom use. The variables of interest were: formal education (stratified as having completed primary education or lower and having completed some secondary education or higher), age, marital status and number of sexual partners in the previous year (classified as one or more than one sexual partner). The type of sexual partner was categorized as regular or casual. A regular sex partner was defined as the person with whom the patient had been sexually involved for more than three months, whether there was an affective link between them or not. Casual partners were defined as those with whom the interviewee had had sexual intercourse without the intention of seeing him/her again or had been sexually involved for less than three months. These definitions were read to interviewees before conducting the relevant part of the interview.

## Results

In all, 466 persons were approached in the waiting area of the clinic; of these, 60% (283) were included in this study based on the inclusion and exclusion criteria. The respondent rate of those persons who met the inclusion criteria was 100%.

The socio-demographic characteristics of the sample are shown on Table 1. At the time of the study, 94.3% of the interviewed persons (267) were undergoing antiretroviral treatment.

**Table 1:** Socio-demographic characteristics of the sample

		n = 283
**Gender**		

	Male	54.8% (155)

	Female	45.2% (128)

**Age**		35.3 ± 10.05

**Marital status**		

	Living with partner	43.8% (124)

	Married	24.7% (70)

	Single	17.0% (48)

	Separated	10.6% (30)

	Widowed	3.2% (9)

	Divorced	0.7% (2)

**Formal education**		

	Completed primary education or lower	50.2% (142)

	Completed some secondary education or higher	31.4% (89)

	None	18.4% (52)

**Literate**		

	Yes	82.9% (232)

	No	17.1% (48)

Regarding knowledge of mechanism of HIV transmission, 93.3% of the patients were aware that HIV could not be transmitted through kisses, and 69.6% were aware that it could not be transmitted through mosquito bites. The mechanisms by which most respondents thought that HIV could be transmitted were: sharing needles when using intravenous drugs (94.3%), having unprotected sex (98.9%), having blood transfusions (96.5%), from the infected mother to the baby during pregnancy or birth (87.3%), from an infected mother to the breastfeeding baby (88.7%), and having anal sex (86.2%). They were aware that that mutual faithfulness (86.9%), using a condom (97.5%), abstinence (73.9%) and using a condom in a vaginal (97.2%) or anal sexual relation (72.4%) were effective ways to prevent HIV transmission.

When asked about sexual partner, 84.8% of the 283 subjects said they had had only one sexual partner in the past year, 15.2% more than one sexual partner, and 4.34% five or more partners. When the distinction was made between regular and casual sexual partners, 85.2% reported having had at least one regular sexual partner and 36.2% at least one casual partner in the previous 12 months. Male respondents had more sexual partners than females (χ2 = 23.67, CI 95%, p = 0.00001), specifically casual partners (χ2 = 13.85, CI 95%, p = 0.00001).

Respondents were asked to classify their sexual partners in the past year as casual or regular and to define their HIV serostatus as positive, negative or unknown. The results are provided in Table 2. In total, 89.6% of the reported sexual casual partners were of an unknown HIV serostatus according to the interviewees, while 58.33% of the reported regular partners were positive according to the interviewees. This difference is statistically significant (χ2 = 333.2, CI 95%, p < 0.0001).

**Table 2:** Reported HIV serostatus according to sexual partner type

Partner HIV serostatus	Regular 44.9% (n = 228)	Casual 55.0% (n = 279)
**Positive**	58.33% (133)	3.58% (10)

**Negative**	32.89% (75)	6.81% (19)

**Unknown**	8.77% (20)	89.60% (250)

There was a difference in condom use in the previous year depending on the type of sexual partner, 81.7% and 87.3% of the patients reported always using a condom with their regular and casual sexual partner, respectively; however, this difference was not statistically significant (OR 1.55, χ^2 ^= 1.34, CI 95%, p = 0.24). Likewise, there was no statistically significant difference in condom use in the past six months according to formal education of the sample (χ2 = 1.12, CI 95%, p = 0.29), gender (χ2 = 2.33, CI 95%, p = 0.09) and the number of sexual partners (OR 0.37, χ2 = 0.00, CI 95%, p = 1). When asked about their most recent sexual relation, 90.4% (255) reported using a condom and 83% (235) reported using a condom in every sexual relation in the previous six months.

At least one reason was given for not using a condom in the past year by 78 people (27.5%). The main reasons mentioned were: "my partner did not want to use a condom" (48.7%); "the condom irritates or makes my partner uncomfortable" (30.8%); and "Condom irritates me or makes me uncomfortable" (29.5%). Two people (2.6%) reported not using a condom in the past year because they were, or their partner was, under the effect of alcohol or another drug. Other reasons mentioned for not using a condom are provided in Table 3.

**Table 3:** Reported reasons for not using a condom in the past year

Reason reported	(n = 78)
My partner did not want to use a condom	48.7%

The condom irritates or makes my partner uncomfortable	30.8%

Condom irritates me or makes me uncomfortable	29.5%

I did not want to use a condom	23.1%

I do not like making love with a condom	21.8%

I do not think it is important since my partner is also HIV positive	16.7%

It was my regular partner	15.4%

We did not have a condom	14.1%

We felt safe	14.1%

My partner loves me	12.8%

My partner thinks he or she cannot get infected with HIV	11.5%

I want to, or my partner wants to get pregnant	10.3%

My partner does not know I am HIV positive	9.0%

We forgot to use a condom	7.7%

We do not have sexual relations with other persons	6.4%

I cannot discuss with my partner the use of condom	6.4%

I think my partner cannot get infected with HIV	2.6%

I was or my partner was under the effect of alcohol	2.6%

## Discussion

Our results show that heterosexual PLHIV recognize condoms as an effective method of preventing HIV transmission and that most of them use condoms. Some of them do not acknowledge its value for the prevention of HIV in anal intercourse. This could be due to the fact that counselling might be focused on vaginal sexual relations.

Most of the women properly identified both pregnancy in an HIV-infected mother and breastfeeding as a possible means of transmission of the virus to the baby. Heterosexual PLHIV recognized mutual faithfulness as a method of preventing transmission of HIV in a larger percentage than that reported by a study among MSM [7].

Self-reported condom use in the past six months and most recent sexual relation is higher than that reported in other studies [9,10]. Condom use consistency in the previous three months among PLHIV in Guatemala in another study [8] is lower than condom use consistency reported by the sample in our study in the previous six months. Nevertheless, the sample in the other study was different from ours: homosexual and bisexual PLHIV were not excluded, they were more recently diagnosed with HIV, they had received less condom counselling, and they were not undergoing antiretroviral therapy. This might explain the differences.

It appears as if a larger percentage of heterosexual PLHIV report using a condom consistently than MSM and commercial sex workers, according to reports by other studies [6,7]. However, these studies have addressed condom consistency in the prior month, which is different from what was addressed in our study. Reported condom use in the most recent sexual relation by MSM in a study done in Guatemala is 80.1% [7], which is less than what was found in our results (90.4%).

Overall, male participants had more sexual partners than female participants, predominantly casual ones. When subjects reported having casual partners, in most cases, they did not know their HIV serostatus. A large percentage, 72% (364), of the total number of sexual partners in the previous year (507) were either HIV negative (94) or of an unknown serostatus (270). Potentially, these HIV-negative partners are at risk of contracting the virus.

When they were asked to cite the reasons for not using a condom in the past year, the main reason given was that their partner did not want to use it. This is a factor that should be addressed during counselling at the clinic, focusing on and strengthening condom use negotiation. In this population, it appears that alcohol and drugs do not affect condom use, as has been found in other studies conducted in other countries [9,10].

Some authors have described changes in sexual behaviour related to the use of antiretroviral therapy [11]. Since most of this sample was currently on antiretroviral therapy, no distinctions could be made. This gives an opportunity for future research focused on recently initiated patients on highly active antiretroviral therapy and modifications in their sexual behaviour.

There is an ongoing debate on the superiority of a self-administered questionnaire over a guided interview [12]. In this study, the interviewer-administered form was preferred, because of the expected low level of education of the population. On the other hand, patients attending the clinic have received counselling on condom use, and this could explain the greater adherence reported by patients.

We think that patients may be conditioned to answer with what they are expected to say. This could be a potential bias leading to an underestimation of condom non-adherence. In fact, more respondents gave reasons for not using a condom, even when they had responded to an earlier question by saying that they always used a condom. Confirmation of condom adherence could be aided with biological markers of new diagnosis of other sexually transmitted infections. Future studies could relate biological markers and reported sexual behaviour.

## Conclusions

This study shows that there is a group of HIV-positive persons who continue to have sexual relations without using condoms, even after counselling. It also contributes to explaining why patients are not using condoms, which can generate new and more focused strategies to promote condom negotiation among partners and emphasize correct and consistent condom use in every sexual relation, including anal intercourse.

Since no socio-demographic variable was associated with inconsistent condom use, we recommend intensive and regular condom counselling for every heterosexual outpatient who attends the clinic. This study did not address social, interpersonal (including disclosure) and psychological factors that might determine condom use among heterosexual PLHIV. These issues should be explored during counselling and can be subjects of further research.

## Competing interests

The authors declare that they have no competing interests.

## Authors' contributions

JJD and MP contributed to the design of the study, performed the statistical analysis and wrote the first draft of the manuscript. IC and CM participated in the design and coordination of the study and critically reviewed the manuscript. All authors read and approved the final manuscript.

## References

[B1] UNAIDS Report UNGASS Guatemala 2010 2010 Guatemala http://www.unaids.org/en/dataanalysis/monitoringcountryprogress/2010progressreportssubmittedbycountries/guatemala_2010_country_progress_report_en.pdf

[B2] Instituto Nacional de Estadística, INE Encuesta Nacional de Condiciones de Vida, ENCOVI-2006 http://www.ine.gob.gt/np/encovi/ENCOVI2006/Resultados_Nacionales.pdf

[B3] USAID HIV/AIDS profile 2009 http://www.usaid.gov/locations/latin_america_caribbean/country/guatemala/

[B4] UNDP Guatemala. Country profile of human development indicators 2010 http://hdrstats.undp.org/en/countries/profiles/GTM.html

[B5] Programa Global de VIH/SIDA Reduciendo la vulnerabilidad al VIH/SIDA en Centroamérica Guatemala: Situación del VIH/SIDA y respuesta a la epidemia 2006 Banco Mundial http://siteresources.worldbank.org/INTHIVAIDS/Resources/375798-1103037153392/CAHIVAIDSGuatemalaFINALSPA.pdf

[B6] Estudio TRaC de VIH/SIDA entre trabajadoras sexuales femeninas en Ciudad de Guatemala, Escuintla, Izabal, Quetzaltenango y Suchitepequez. Segunda Ronda. División de investigación de PSI 2008 http://www.pasca.org/sites/default/files/07_gt_trac_tsf.pdf

[B7] Estudio TRaC de VIH/SIDA Hombres que tienen sexo con otros Hombres en Guatemala, Quetzaltenango, Escuintla, Suchitepéquez e Izabal. Segunda Ronda. División de investigación de PSI 2009 http://www.pasca.org/sites/default/files/GUA_TRaC_2009_HSH_final_6sept.pdf

[B8] Samayoa B Anderson MR O'Sullivan LF Patricia K Pacheco A Matos A Reyes DA Setru S Arathoon E Does HIV VCT reduce risk behavior? An observational study in Guatemala City Current HIV Research 2010 14 121 126 10.2174/157016210790442696 20163346

[B9] Dave SS Stephenson J Mercey DE Panahmand N Jungmann E Sexual behavior, condom use, and disclosure of HIV status in HIV infected heterosexual individuals attending as inner London HIV clinic Sex Trans Infect 2006 14 117 120 10.1136/sti.2005.015396 PMC2564680 16581734

[B10] Clark RA Kissinger P Bedimo AL Dunn P Albertin H Determination of factors associated with condom use among women infected with human immunoddefficiency virus Int J STD AIDS 1997 14 229 233 10.1258/0956462971919976 9147155

[B11] Dukers NH Goudsmit J de Wit JB Prins M Weverling GJ Coutinho RA Sexual risk behaviour relates to the virological and immunological improvements during highly active antiretroviral therapy in HIV-1 infection AIDS 2001 14 369 378 10.1097/00002030-200102160-00010 11273217

[B12] Crepaz N Marks G Towards an understanding of sexual risk behavior in people living with HIV: a review of social, psychological, and medical findings AIDS 2002 14 135 149 10.1097/00002030-200201250-00002 11807297

